# Cerebellar anatomical alterations and attention to eyes in autism

**DOI:** 10.1038/s41598-017-11883-w

**Published:** 2017-09-20

**Authors:** Charles Laidi, Jennifer Boisgontier, M. Mallar Chakravarty, Sevan Hotier, Marc-Antoine d’Albis, Jean-François Mangin, Gabriel A. Devenyi, Richard Delorme, Federico Bolognani, Christian Czech, Céline Bouquet, Elie Toledano, Manuel Bouvard, Doriane Gras, Julie Petit, Marina Mishchenko, Alexandru Gaman, Isabelle Scheid, Marion Leboyer, Tiziana Zalla, Josselin Houenou

**Affiliations:** 1 UNIACT, Psychiatry Team, Neurospin Neuroimaging platform, CEA Saclay, Gif-Sur-Yvette, France; 2 INSERM Unit U955, Team 15 “Translational Psychiatry”, Institut Mondor de Recherche Biomédicale, Créteil, France; 3 Fondation Fondamental, Créteil, France; 4 Pôle de Psychiatrie, Assistance Publique–Hôpitaux de Paris (AP-HP), DHU PePsy, Hôpitaux Universitaires Mondor, Créteil, France; 5 Cerebral Imaging Center, Douglas Mental Health University, McGill University, Montréal, Canada; 6 Departments of Psychiatry and Biological and Biomedical Engineering, McGill University, Montreal, Canada; 7 UNATI, Neurospin, CEA, Paris Saclay University, Gif-Sur-Yvette, France; 8 Service de psychiatrie de l’enfant et de l’adolescent, Assistance Publique–Hôpitaux de Paris (AP-HP), Hôpital Robert Debré, Paris, France; 9 Institut Pasteur, Human Genetics and Cognitive Functions Unit, Paris, France; 10 Neuroscience, Ophthalmology, and Rare Diseases (NORD), Roche Pharma Research and Early Development, Roche Innovation Center Basel, F. Hoffmann-La Roche Ltd., Basel, Switzerland; 11 Charles Perrens Hospital, Autism Expert Center, Bordeaux, France; 12 Laboratoire de Linguistique Formelle UMR 7110, Centre National de Recherche Scientifique, Université Paris Diderot, Paris, France; 13 Institut Jean Nicod (UMR 8129), CNRS, Ecole Normale Supérieure & PSL Research University, Paris, France; 14 Faculté de Médecine, Universite Paris Est, Créteil, France; 15 Institut Roche, Roche Pharmaceuticals, Boulogne-Billancourt, France

## Abstract

The cerebellum is implicated in social cognition and is likely to be involved in the pathophysiology of autism spectrum disorder (ASD). The goal of our study was to explore cerebellar morphology in adults with ASD and its relationship to eye contact, as measured by fixation time allocated on the eye region using an eye-tracking device. Two-hundred ninety-four subjects with ASD and controls were included in our study and underwent a structural magnetic resonance imaging scan. Global segmentation and cortical parcellation of the cerebellum were performed. A sub-sample of 59 subjects underwent an eye tracking protocol in order to measure the fixation time allocated to the eye region. We did not observe any difference in global cerebellar volumes between ASD patients and controls; however, regional analyses found a decrease of the volume of the right anterior cerebellum in subjects with ASD compared to controls. There were significant correlations between fixation time on eyes and the volumes of the vermis and Crus I. Our results suggest that cerebellar morphology may be related to eye avoidance and reduced social attention. Eye tracking may be a promising neuro-anatomically based stratifying biomarker of ASD.

## Introduction

Autism spectrum disorder (ASD) is a neurodevelopmental condition affecting 1 in 68 children^1^. Its symptoms include impaired communication and social interactions along with restricted or repetitive behaviors.

Previous studies in children and adolescents with ASD have implicated cerebellar morphological abnormalities in ASD^2,3^ and its symptoms. However, cerebellar morphology has not been thoroughly investigated in adults with ASD.

The cerebellum represents 10% of total brain volume but contains more than 50% of its neurons^4,5^. Also referred to as the “little brain”, the cerebellum expanded more than the neocortex during the evolution of apes^6^ and is notably larger in humans in comparison with other higher primates. Since Leiner *et al*.^7^ first proposed in 1986 that the cerebellum was implicated in non-motor functions, substantial evidence has demonstrated that it is involved in a broad range of cognitive functions^8^ including emotion regulation and social cognition^9,10^. Recent neuroimaging^9^ and lesion studies^11^ have proposed functional topography of the cerebellum such that the cerebellum can be divided in regions based on their connectivity with the sensorimotor vs. multimodal association cortices^12^. The posterior lobe of the cerebellum is involved in cognition and connected to associative regions such as the prefrontal cortex and the superior temporal region, whereas the anterior cerebellum likely modulates sensory-motor cortex activity. Therefore, it has been proposed that the cerebellum detects, prevents and corrects mismatches between intended and perceived outcomes of an organism’s interactions with the environment^13^.

Several cerebral regions are involved in the pathophysiology of ASD but abnormalities in the cerebellum are among the most reproducibly reported alterations in this disorder^14^. Neuropathological studies show a decrease in the number, size and density of Purkinje cells in the cerebellar cortex of individuals with ASD (see Fatemi *et al*.^15^ for full review) and genetic studies demonstrate high cerebellar co-expression of genes associated with ASD^16^. Recently, Wang *et al*.^17^ proposed that early perinatal alterations of the cerebellum contribute to the pathogenesis of ASD.

Cerebellar morphology has been investigated in individuals with ASD^2,3,18–23^ and differences in the volume, as compared to healthy people, of Crus I^3^, cerebellar vermis^19,23^ and anterior cerebellum^24–26^ have been reported.

While the anterior part is connected to sensory-motor cortex, and has been more extensively studied in individuals with ASD due to their known sensory-motor impariments, social aspects of the cerebellum are still understudied in autism. The posterior cerebellum is known to be involved in the recognition of facial expression (impaired in ASD)^27^ in patients with ischemic posterior cerebellar lesions or with cerebro-cerebellar degeneration^28^. In addition, the posterior cerebellum and in particular Crus I are involved in social cognition^13,28^ through cortico-cerebellar connections with the posterior superior temporal sulcus^29^, which are disrupted in ASD^25^.

Deficits in recognition of facial expressions and social perception in ASD may be mediated by abnormal eye contact^30^. Eye contact abnormalities are a key feature of ASD^31^ and are part of the diagnostic criteria within the DSM-5. Eye-tracking is a method for recording eye movements using an infrared camera, in order to estimate direction of gaze. It has been proposed that eye-tracking may be used as a biomarker to assess the efficacy of potential treatments for ASD^32,33^ or for early detection of eye contact abnormalities in children who may be later diagnosed with ASD^31^.

The aim of our study was to investigate the morphology of the cerebellum in a sample of adult individuals with high-functioning ASD and to explore the relationship between a key eye-tracking outcome (fixation time allocated to the eye region during an emotion recognition task) and the morphology of the cerebellar lobules involved in social cognition (Crus I, vermis). To our knowledge, no study to date has investigated the cerebellar morphology abnormalities in adults with ASD and their association with fixation time on the eye region. Based on previous literature and the large number of cerebellar sub-volumes, we focused our analyses on three regions of interest: the anterior cerebellum, Crus I (bilateral) and the vermis.

## Methods and Material

### Participants

We recruited individuals with ASD without intellectual disability and controls from the Mondor University Hospital (Créteil, France). Diagnoses were confirmed by trained clinicians, the Autism Diagnostic Interview-Revised (ADI-R) and the Autism Diagnostic Observation Schedule (ADOS). Controls were recruited through advertisements in the local press. Non inclusion criteria were: an age below 18, a personal history of intellectual disability or neurological disorder and a substance use disorder except for tobacco (all subjects recruited had no history of alcohol abuse or dependence). Controls with a personal history of axis I disorder or with a first-degree relative with schizophrenia, schizoaffective, or bipolar disorder were not included. All individuals with ASD and controls had a minimum full scale IQ of 70.

In order to increase the statistical power of our analyses, we also included individuals with ASD and controls from the Autism Brain Imaging Data Exchange (ABIDE I and II) samples. All details on inclusion criteria are available on the ABIDE Website (http://fcon_1000.projects.nitrc.org/indi/abide/) and have been published elsewhere^34,35^. We selected indviduals with ASD and controls with a minimum full scale IQ of 70. We excluded subects with an age below 18.

We only included individuals with ASD for whom the entire volume of the cerebellum was available, resulting in the exclusion of 37 subjects (19 individuals with ASD and 18 controls). For ABIDE samples, in order to limit the site effect, we selected 5 sites that included more than ten individuals with ASD and ten controls matching our inclusion criteria (adults for whom the entire volume of the cerebellum was available).

**Figure 1:**
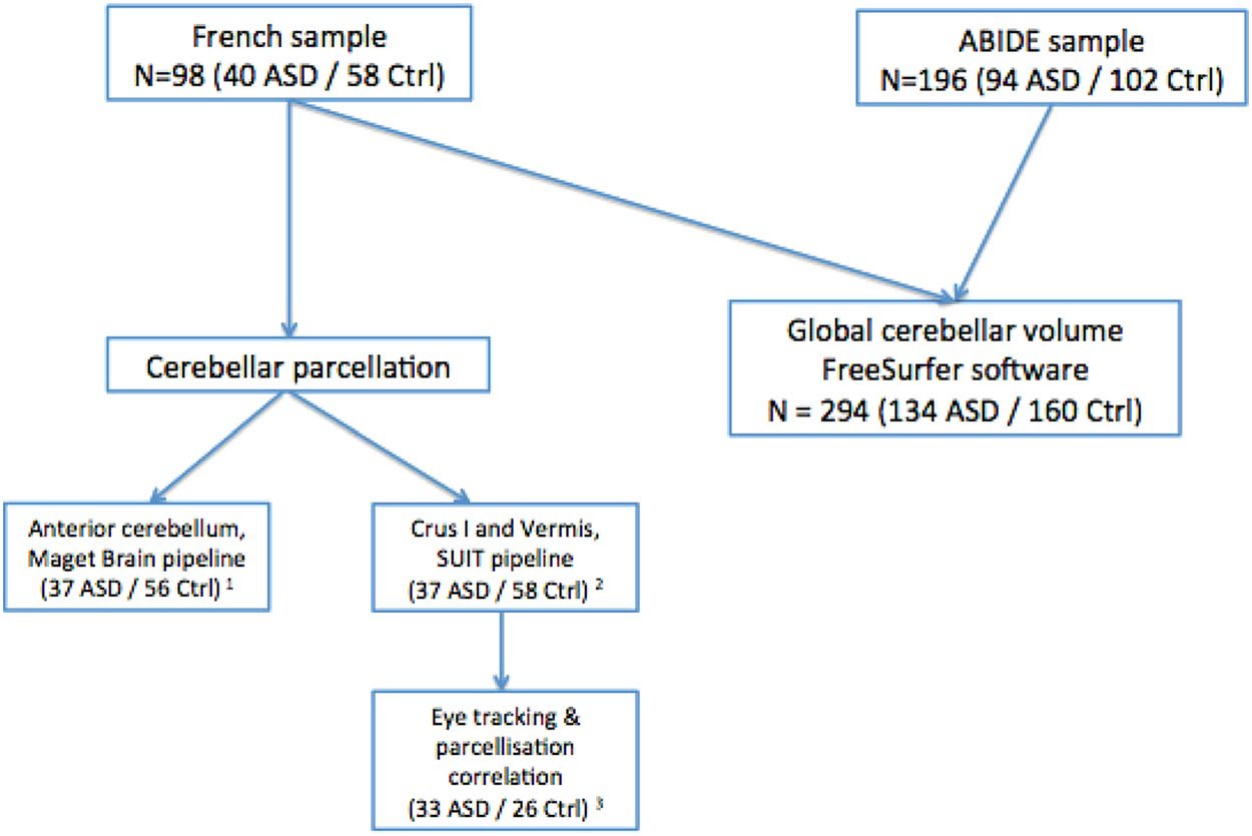
Flow chart. ASD: indviduals with ASD. Ctrl: healthy controls. 1 = From the initial sample (40 ASD and 58 HC), five subjects (two controls and three individuals with ASD) were excluded after quality control. 2 = From the initial sample (40 ASD and 58 HC), three subjects (three individuals with ASD) were excluded after quality control. 3 = From the sample included in the SUIT pipeline analysis (37 ASD and 58 HC), 33 individuals with ASD and 26 HC underwent our eye-tracking assessment and where included in the analysis.

We included in our study 134 individuals with ASD and 160 controls aged from 18 to 64 years. Participants are described in Table 1 and in Fig. 1. We were not able to report all IQ of controls in Table 1 because this information was not collected for every subject. However all controls had a minimum IQ of 70 as assessed by the Kaufman Brief Intelligence Test-2 (KBIT2) in the Barrow Neurological Institute and the National Adult Reading Test (NART) in Créteil (France).

**Table 1:** Demographic and clinical characteristics of participants.

	ASD (n = 134)	Control (n = 160)	Chi-2/t-test
Mean age (SD)	28 (10)	30 (11)	Student t-test; p = 0.2
Sex; Men (%)	117 (87)	133 (83)	Chi-2; p = 0.5
Site	Créteil (n = 40)^†^	Créteil (n = 58)^†^	—
	NYU^1^ (n = 12)	NYU^1^ (n = 23)	—
	USM^2^ (n = 33)	USM^2^ (n = 21)	—
	CAL^3^ (n = 13)	CAL^3^ (n = 15)	—
	BNI^4^ (n = 22)	BNI^4^ (n = 25)	
	IU^5^ (n = 14)	IU^5^ (n = 18)	
Mean ADOS scores (SD)	12,3 (4)*	—	
Mean full scale IQ (SD)	104 (16)**	N/A	

^1^NYU, New York University, ABIDE I sample.

^2^USM, University of Santa Monica, ABIDE I sample.

^3^CAL, California Institute of Technology, ABIDE I sample.

^4^BNI, Barrow Neurological Institute, ABIDE II sample.

^5^IU, Indiana University, ABIDE II sample.

N/A: not available.

^†^40 individuals with ASD and 58 controls were included in the global segmentation analysis. After quality-check, 37 individuals with ASD and 56 controls were included in the anterior lobe analysis; 37 individuals with ASD and and 58 controls were included in the Crus I/Vermis analysis. In the eye-tracking analysis, 33 indviduals with ASD and 26 controls were included (See Supplementary Material 2 for full Table).

*Data for BNI missing (different versions of the ADOS scores (total of communication and social interaction subscores) that could not be computed in the same mean).

**Data missing for 31 individuals with ASD (but global IQ > 70) or IQ > 85 (−1 DS) assessed with the Kaufman Brief Intelligence Test-2 (KBIT2) in the BNI center and the National Adult Reading Test (NART) in Créteil (France).

A sub-sample of 59 subjects, recruited in Créteil (France), underwent in addition an eye tracking protocol. Demographic and clinical characteristics of these participants are reported in Supplementary Material 2.

All methods were carried out in accordance with relevant guidelines and regulations: All experimental protocols that included French individuals with ASD were approved by local ethics committee (CPP Ile-de-France IX). Written informed consent was obtained from all participants after receiving a full description of the study.

### MRI acquisition

All subjects recruited in France underwent an MRI acquisition on a 3T Siemens Magneton Tim Trio, with a standard twelve channels head coil, at the Neurospin neuroimaging center (CEA Saclay, 91, France). We used a 3DT1 MPRAGE sequence (160 slices, TR = 2200 ms, TE = 5.16 ms, FOV = 256 × 256 mm^2^, voxels of 1 × 1 × 1.1 mm). Acquisition parameters from the ABIDE dataset are described elsewhere (http://fcon_1000.projects.nitrc.org/indi/abide/).

### Cerebellum global segmentation and lobular parcellation pipeline

A flow chart describing the analyzed samples is reported in Fig. 1.

First, we used FreeSurfer 5.3.0 software to perform global segmentation of cerebellar left and right cortical and white matter volumes. The automated procedure for volumetric measures of subcortical structures and cerebellum has been previously described^36,37^. This technique, performing segmentation and labeling of brain structures, is reported to be as effective as manual tracing^36^.

Second, to better understand the cerebellar structures in individuals with ASD, we performed a lobular parcellation of the cerebellum.

We used two different methods (MAGeT Brain^38^ for the anterior lobe and SUIT^39^ for the vermis and Crus I) to perform cerebellar parcellation. We performed parcellation of the anterior cerebellum using the MAGeT Brain pipeline because this method is more accurate in this particular region compared to the SUIT algorithm. Park *et al*.^38^ compared both methods with manual segmentation, using high resolution MRI, in the anterior lobe of the cerebellum and found significant increased Kappa values in the anterior lobe (lobules I to IV and lobule V) with MAGeT Brain compared to SUIT. In addition, Park *et al*. also reported inaccuracies in the isolation map of the SUIT pipeline located in the anterior cerebellum.

Prior to the cerebellar parcellation with the MAGeT brain pipeline, we preprocessed (https://github.com/CobraLab/minc-bpipe-library) the T1 images and performed N4 correction, cutneck and registration to the MNI space using the Advanced Normalization Tools (ANTs) software^40^. The MAGeT brain^41^ pipeline uses five high definition templates manually labeled by two expert raters. These templates are first tuned to the specificities of our acquisitions from the generation of multiple anatomical segmentations of representative images selected within our sample. These segmentations become dedicated templates. Each subject’s brain is then registered to each of these templates, which takes into account the neuroanatomical variability within a given population. This pipeline has previously been applied to the cerebellum^38^ and is comparable to the gold standard manual segmentation. Within our sample, we selected 21 templates (ten controls and eleven individuals with ASD) with a similar sex ratio and mean age.

We performed the parcellation of the cerebellar vermis (vermal portion of the posterior lobe of the cerebellum) and Crus I with the SUIT^39^ pipeline as the MAGeT Brain atlas does not provide a label for the cerebellar vermis. The Crus I label of the MAGeT Brain atlas is merged with the vermal part of Crus I. We examined the volume of the vermis and of Crus I separately as it is possible that changes in these two regions differ depending on diagnosis. Since a segmentation of the vermis is not proposed in MAGeT Brain, the anatomical landmarks of Crus I differ with SUIT. Park *et al*.^38^ did not compare the two methods for Crus I. In addition, we saw a discrepancy in the segmentation of Crus I using MAGeT Brain pipeline. We found an over or an under segmentation of Crus I, with the inferior part of Crus I being labeled as Crus II or the superior part of Crus II being labeled as Crus I. This defect was not observed when using the SUIT algorithm (See Supplementary Material 1B).

Overall, we used the SUIT pipeline for the segmentation of the vermis and Crus I because (i) SUIT was the only method providing segmentation of the vermis and (ii) we observed a defect in the segmentation of Crus I using the MAGeT Brain pipeline (whereas SUIT accurately segmented this region, see Supplementary Marterial 1).

In the SUIT pipeline^39^, cerebellar structures are automatically isolated from the cerebral cortex based on an anatomical image. This toolbox provides an automated cortical segmentation of the cerebellum, using a probabilistic atlas to assign locations to different cerebellar lobules.

**Figure 2:**
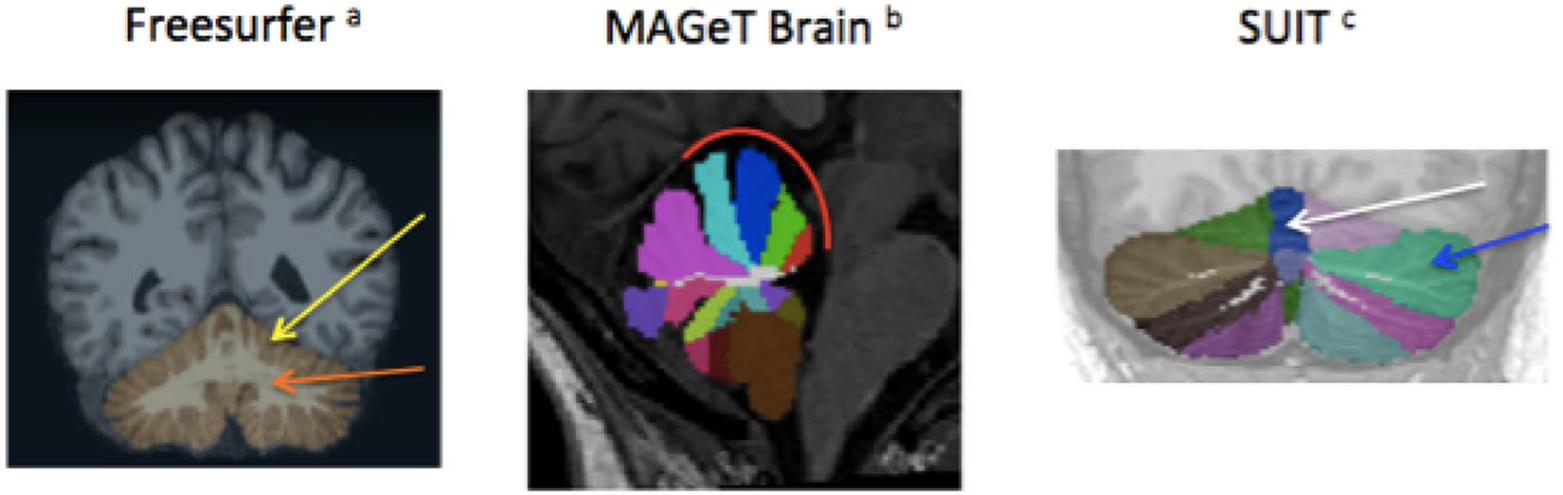
Cerebellar segmentation. (**a**) Fischl *et al*.^36^. (**b**) Park *et al*.^38^. (**c**)Diedrichsen *et al*.^39^.

Cerebellar segmentation and parcellation are represented in Fig. 2.

### Data processing and quality control

For the FreeSurfer segmentation analysis (segmentation of cerebellar cortical and white matter), an examiner (CL), blind of the diagnosis, visually inspected the full coverage of the cerebellum and all segmentations to ensure quality in the segmentation. Six screenshots centered on the cerebellum were inspected: two on sagittal slices (10 slices away from the midline on the left and the right side of the brain), two on axial slices (centered on the midline) and two on coronal slices (centered on the midline). We excluded subjects with motion artifacts, with no full coverage of the cerebellum, with a significant segmentation defect in the cerebellum (either tissues mislabelled as cerebellum or cerebellar tissue not labeled as cerebellum) or with a significant anatomical abnormality in the posterior fossa (such as a cyst). We ensure that this quality check was efficient by visually inspecting the 3D volumes of 30 subjects as previously described^37^.

We excluded eight subjects because of a defect (non cerebellar volume segmented) in the cerebellar segmentation using the FreeSurfer software (five individuals with ASD and three controls), two controls due to a cyst in the posterior fossa and one subject due to significant motion artifacts.

We thus performed FreeSurfer segmentation on a total of 134 individuals with ASD and 160 controls (Table 1).

For the parcellation analysis, an examiner (CL), blind of the diagnosis, visually inspected the 3D volumes for each subject of the parcellated cerebellum in axial, coronal and sagittal views.

We only performed cerebellar parcellation in subjects from France (n = 40 individuals with ASD and n = 58 controls) and not in the ABIDE sample (see supplementary Material 1A).

After visually inspecting each cerebellar parcellation, five subjects were excluded from the MAGeT Brain analysis on the anterior cerebellum (three individuals with ASD and two controls), and three subjects were excluded from the SUIT analysis on Crus I and the vermis (three individuals with ASD). Subjects were excluded because of inaccurate segmentation of non-cerebellar tissue or because the anterior lobe, Crus I or the vermis were not accurately segmented (Supplementary Material 1B).

### Eye-tracking acquisitions and data processing

**Figure 3:**
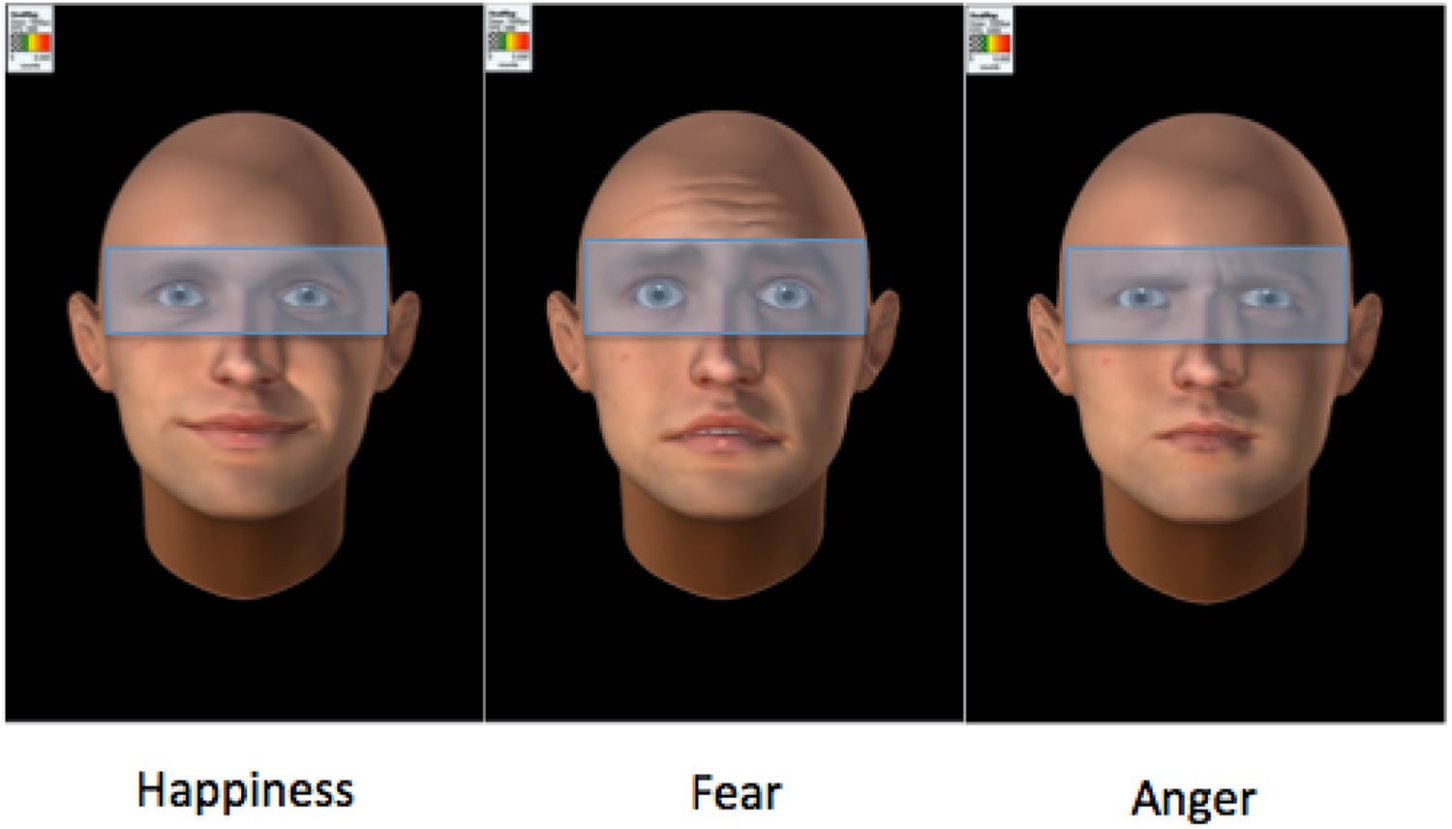
Emotional faces showed during the eye-tracking task. Translucent blue rectangle: region of interest defined to measure the fixation time on eyes (ms) during the eye-tracking task. Avatar were created with FACSGen software developped by the Swiss Center for Affective Neuroscience (Modified from Roesch *et al*.^77^ with copyright holder permission).

Subjects recruited in France underwent a free-viewing eye-tracking task using a screen-based (23”, resolution set at 1920 × 1080 pixels) Tobii TX300 device (Tobii Technology, Stockholm, Sweden), allowing gaze acquisition at 300 Hz. After a five-point calibration, 18 dynamic avatar faces (Fig. 3) were presented, each remaining for three seconds and followed by a constant interstimulus interval (20 s). The faces randomly expressed dynamically fear, anger or happiness.

We extracted the fixation time (in milliseconds) on eye regions for each of the three emotions. Our variable of interest was the average of this fixation time of the three conditions. Further details on the eye-tracking procedure are provided in Supplementary Material 3. See Fig. 1 (flow-chart) for the representation of subjects enrolled in both MRI and eye-tracking analyses.

### Statistical analyses

We used a Chi-square test to test for significant differences in the proportion of males and females and a Student t-test to compare ages between individuals with ASD and controls (Table 1 and Supplementary Material 3).

To compare the global size of the left and right cerebellum (grey or white matter) between individuals with ASD and controls, we performed an ANCOVA, with (i) age and intracranial volumes as covariates and (ii) sex, diagnosis (fixed effect) and site of inclusion (random effect) as cofactors. We repeated the same analysis in the sample recruited in France, without considering site of inclusion as a covariate.

In order to examine diagnostic related differences in volume in the cerebellar sub-regions (left/right anterior cerebellum, left/right Crus I and vermis) we performed an ANCOVA, with (i) the ratio between cerebellar sub-volume and the global cerebellar volume as the dependent variable, (ii) sex and diagnosis as cofactors (fixed effect) and (iii) age as a covariate. Results were corrected for multiple comparisons (five tests) using a False Discovery Rate (FDR) – Benjamini Hochberg correction.

To study the relationship between the fixation time allocated to the eye region during an emotional condition and cerebellar sub-volumes (left/right anterior cerebellum, left/right Crus I and vermis) in the individuals with ASD and the control groups, we performed linear regressions with (i) the ratio between cerebellar sub-volume and the global cerebellar volume as the dependent variable, (ii) sex as a cofactor (fixed effect) and (iii) age and fixation time on eyes as covariates. Analyses were carried out separately in individuals with ASD and controls. As our paper focused on the cerebellar anatomy and the associated clinical features, we chose to include cerebellar sub-volumes as a dependant variable and fixation time on eyes as covariate. However, interverting both variables (i.e considering fixation time on eyes as a dependant variable and cerebellar sub volumes as covariate) did not change our results. Results were corrected for multiple comparisons (six tests) using a False Discovery Rate (FDR) – Benjamini Hochberg correction.

We present both uncorrected (presented as “p” value) and corrected p values (presented as p_corrected_).

Before performing pairwise comparisons (t-tests) between individuals with ASD and controls, the following assumptions were checked: (i) standardized residuals were normally distributed, as assessed by Shapiro–Wilk test (P > 0.05) and a QQ-plot; (ii) homoscedasticity and homogeneity of variances, as assessed by visual inspection of a scatterplot and Levene’s Test of homogeneity of variance. Outliers were defined as cases with standardized residuals greater than 4 standard deviations and were not included in the analysis.

Because there is a higher rate of ASD diagnosis in males compared to females^42^, and that it has been proposed that males and females with autism may have different forms of autism, we included sex as a covariate in our statistical models and tested a sex x diagnosis interaction.

We conducted several post-hoc analyses. We repeated the main analyses by excluding females and only considering a male sub-sample. To test the effect of ADOS scores (total of communication and social interaction subscores) on cerebellar morphology in the patient group, we conducted a linear regression considering (i) the ratio between cerebellar sub-volume and the global cerebellar volume as the dependent variable, (ii) sex as a cofactor and (iii) age and ADOS as covariates. To test the effect of ADOS scores on fixation time to the eyes in the patient group, we conducted a linear regression considering (i) the fixation time to the eyes as a covariate, (ii) sex as a cofactor and (iii) age and ADOS as covariates.

To test the effect of IQ on our results in the ASD group, we conducted a linear regression considering (i) the ratio between cerebellar sub-volume and the global cerebellar volume as the dependent variable, (ii) sex as a cofactor and (iii) age and IQ as covariates. In our model testing the effect of fixation time to the eyes on cerebellar volume, we included IQ as a covariate in order to study the potential confounding effect of IQ. We included intra cranial volume (ICV) as a covariate in our parcellation analyses to account for the effect of ICV.

We conducted all statistical analyses with SPSS version 20.0 (IBM) at the exception of the FDR correction performed with python statsmodels open source library^43^.

## Results

### Demographic and clinical characteristics

Demographics and clinical characteristics of the study sample are described in Table 1. There were no significant differences in mean age or sex ratio between individuals with ASD and controls.

### Cerebellar grey and white matter volumes

One-hundred thirty-four individuals with ASD and 160 controls were included in this initial analysis. We found no significant difference between the two groups for left and right cerebellar cortical volumes and no significant difference in left and right white matter volume. There was no significant effect of sex in our models. We did not find age x diagnosis or sex x diagnosis interactions for any of the four volumes considered. We found the same results within the sample recruited in France (in Créteil, see Table 1): there was no significant difference between individuals with ASD and controls for left and right cortical volumes and no significant difference in left and right white matter volume. We did not find any significant effect of sex in our model, or any age x diagnosis or sex x diagnosis interactions for the four volumes considered.

### Anterior lobe

Thirty-seven individuals with ASD and 56 controls recruited in France were included in this analysis. We found a significant difference for the volume of the right cerebellar anterior lobe (Table 2). This lobe was smaller in individuals with ASD compared to controls (F = 7.45; p = 0.008; p_corrected_ = 0.04). We did not find any significant difference between the two groups for the left anterior lobe (F = 0.24; p = 0.627; p_corrected_ = 0.701). There was no significant effect of sex and no sex x diagnosis interaction in our models.

**Table 2:** Estimated volume (percentage of the total cerebellar cortical volume) of cerebellar left and right anterior lobe in individuals with ASD compared with healhty controls.

Structure	Side	ASD	HS	ANCOVA			
		M (CI 95%)	M (CI 95%)	p	Corrected p value (FDR)	df	Effect-size for **diagnosis**, gender, age (Eta-squared effect size)
Anterior lobe	Right	0.064 (0.062–0.066)	0.067 (0.065–0.068)	0.008 *	0.04	1	**0**.**077**; <0.001; 0.016
	Left	0.065 (0.063–0.067)	0.066 (0.064–0.068)	0.627	0.701	1	**0**.**03**; 0.006; 0.04

ASD, individuals with autism spectrum disorder; HS, healthy subjects.

Df, degrees of freedom.

M, mean; CI, confidence interval; ANCOVA with age, sex as covariates.

Means are calculated with the following covariate: age = 30.71.

*p values < 0,05.

### Crus I and cerebellar vermis

Thirty-seven individuals with ASD and 58 controls recruited in France were included in this analysis.

We did not find any significant differences in volume between individuals with ASD and controls in the left Crus I (F = 0.35; p = 0.558; p_corrected_ = 0.701), right Crus I (F = 0.15; p = 0.701; p_corrected_ = 0.701) or the vermis (F = 1.00; p = 0.320; p_corrected_ = 0.701). There was no significant effect of sex or sex x diagnosis interaction for left/right Crus I. However we found an increased size of the vermis in females compared to males (F = 8.42; p = 0.005) but no sex x diagnosis interaction (F = 0.033; p = 0.856).

### Correlation between eye tracking and cerebellar morphology

Fifty-nine subjects (33 individuals with ASD and 26 controls, for which we had both MRI and eye-tracking measures) were included in this analysis. Demographic and clinical characteristics of this sample are described in Supplementary Material 2. There was no significant difference of IQ, intracranial volume or sex ratio between ASD and controls. Mean fixation time on the eyes was lower for individuals with ASD compared to healthy controls although these differences were not statistically significant (Supplementary Material 2).

Within the ASD group, we found a significant negative correlation between the vermal volume and the fixation time on eyes (Standardized regression coefficient (SRC) = −0.47; p = 0.009; p_corrected_ = 0.018), and a significant positive correlation between the volumes of left (SRC = −0.50; p = 0.007; p_corrected_ = 0.018) and right (SRC = 0.49; p = 0.008; p_corrected_ = 0.018) Crus I and the fixation time on the eye region. Results are reported in Table 3. In the control group, we found no significant correlation between the fixation time on eyes and left Crus I (Standardized regression coefficient (SRC) = −0.123; p = 0.57; p_corrected_ = 0.696), right Crus I (SRC = −0.117; p = 0.58; p_corrected_ = 0.696) or the vermis (SRC = −0.070; p = 0.75; p_corrected_ = 0.75). Scatter plots for individuals with ASD and controls are presented in Supplementary Material 4. There was no significant effect of sex in our models.

**Table 3:** Multiple regression analyses - Attention to eyes predicts cerebellar sub-volumes in individuals with ASD.

Structure	Attention to eyes (ms), Age, Gender		
	Standardized regression coefficient	p-value	FDR corrected p-value - Attention to eyes (ms)
Left Crus I	**0**.**50**; −0.01; 0.14	**0**.**007**; 0.92; 0.42	**0**.**018**
Right Crus I	**0**.**49**; −0.01; 0.05	**0**.**008**; 0.93; 0.76	**0**.**018**
Vermis	−**0**.**47**; 0.11; 0.13	**0**.**009**; 0.49; 0.42	**0**.**018**

Each sub-volume (Left Crus I, Right Crus I and the Vermis) is defined as the percentage of the total cerebellar cortical volume.

### Post-hoc analyses

There were no significant correlations between the anterior cerebellum, Crus I or the vermis and the ADOS scores. In addition, there was no correlation between the fixation time on the eyes and the ADOS scores. There was no significant correlation between age and fixation time on the eyes.

We performed additional analyses to test for confounding factors.

The prevalence of ASD is higher in males than females and there is an ongoing debate to explain these differences^44^. Running our analyses while considering only the males of our sample did not change our results. All positive results remained significant: cerebellar right anterior lobe was smaller in individuals with ASD compared to controls (F = 9.142; p = 0.004); within the ASD group, there was a significant negative correlation between the vermal volume and the fixation time on eyes (Standardized regression coefficient (SRC) = −0.50; p = 0.014), and a significant positive correlation between the volumes of left (SRC = 0.55; p = 0.006) and right (SRC = 0.48; p = 0.021) Crus I and the fixation time on the eye region.

All subjects in our sample had a normal IQ (defined as full scale IQ > 70). In the ASD group, we found no effect of IQ on left/right Crus I or on vermis volumes nor on fixation time on eyes. Correlations between cerebellar sub-volumes and the fixation time on eyes remained significant when including IQ as a covariate in our multiple linear regression models.

We included the intra-cranial volume (ICV) as a covariate in our parcellation analyses, which did not change our results. All positive results remained significant: cerebellar right anterior lobe was smaller in individuals with ASD compared to controls (F = 7.340; p = 0.008); within the ASD group, there was a significant negative correlation between the vermal volume and the fixation time on eyes (Standardized regression coefficient (SRC) = −0.47; p = 0.009), and a significant positive correlation between the volumes of left (SRC = 0.40; p = 0.025) and right (SRC = 0.40; p = 0.022) Crus I and the fixation time on the eye region.

## Discussion

We aimed to investigate the morpholgy of the cerebellum in ASD by performing both global cerebellar segementation and parcellation of the cerebellum. While we did not observe any difference in global cerebellar volumes between groups, the regional analyses yielded a decrease of the volume of the right anterior cerebellum in subjects with ASD compared to controls. Moreover, we found significant correlations between fixation time on eyes and volumes of the vermis and Crus I. Our results suggest that there may be subtle morphological abnormalities in the cerebellum of individuals with ASD and that cerebellar morphology is associated with fixation time on the eye region in individuals with ASD.

Crus I is located in the posterior lobe of the cerebellum (lobule VII). This region is connected to two regions involved in ASD: the superior temporal sulcus^25,45,46^ and the prefrontal lobe^47^. The volume of Crus I has been investigated in ASD primarily in pediatric populations. Two recent studies found opposing results: D’Mello *et al*. reported^2^ decreased volume of Crus I, whereas Sussman^3^ found increased volume of Crus I in individuals with ASD. Interestingly, Rojas *et al*.^48^ found in a sample of adult individuals with ASD a smaller volume of Crus I.

We did not find a difference in Crus I volume between ASD and individuals with ASD. However, we found that that the volume of Crus I was positively correlated with the fixation time to eyes as measured with an eye-tracking device. These results suggest that the volume of Crus I may be smaller in individuals with ASD with eye contact impairments and could explain the discrepant findings in neuroimaging studies that include heterogeneous individuals in the broad spectrum of ASD.

The superior temporal sulcus is involved in social cognition and theory of mind. Both abnormal activations in fMRI studies and structural modifications have been reported in individuals with ASD^46^. In our opinion, this result is of relevance because the Crus I region of the cerebellum and the superior temporal sulcus are known to be connected. This connectivity has been studied with functional^25^ and diffusion MRI^45^ studies. Igelström *et al*.^25^ reported abnormal connectivity between these two regions in individuals with ASD. Our results suggest that the fixation times on the eye region, a potential biomarker of ASD, may explain the anatomical variability in two regions (Crus I and the superior temporal sulcus) connected together and previously linked to social cognition and the pathophysiology of ASD.

The cerebellar vermis is the unpaired median portion of the cerebellum that connects the two hemispheres. Several lines of evidence suggest that the vermis (referred to as the “limbic cerebellum”) is involved in social and affective processing (see Chapter 22 - *Cerebellar Connections with Limbic Circuits*: *Anatomy and Functional Implications* in *Handbook of the Cerebellum and Cerebellar Disorders | Mario Manto | Springer* n.d for full review). Stimulation of the vermis influences the firing patterns in limbic structures such as the hippocampus and the amygdala^49–51^. Cerebellar vermis activations have been reported in a wide variety of fMRI studies using emotion regulation and emotion processing paradigms^49,52^. Acquired lesions of the vermis in children with cerebellar tumors have been associated to dysregulation of affect^53^.

The size of the vermis has been repeatedly investigated in individuals with ASD. Three studies have found a smaller cerebellar vermis in individuals with ASD^20,23^ whereas other authors did not report significant differences^54,55^. Interestingly, in two studies^56^, the vermal volume of lobule VI–VII was either hypo or hyperplastic in two different subgroups of individuals with ASD. While we did not find a difference in size in the vermis of individuals with ASD as compared to controls, we found a negative correlation between vermal size and fixation time on the eye region in the ASD group. Our results are in line with those of Courchesne *et al*.^56^ suggesting that there is heterogeneity in the morphology of the vermis in individuals with ASD. We propose that eye tracking may help to disentangle the heterogeneity of ASD in neuroimaging studies.

In individuals with ASD, we found a positive correlation between the size of the Crus I region and fixation time allocated to the eye region. In addition, we found a negative correlation between the size of the vermis and fixation time to the eyes. These results suggest that patients with altered fixation time to the eyes tend to have a smaller Crus I volume and an increased size of the vermis.

Kansal *et al*.^57^ studied the association between cerebellar sub volumes and a broad range of cognitive functions and reported associations between cognitive dysfunctions and cerebellar atrophy, which is in line with our results within the Crus I lobule. Patterns of lobular variance in the cerebellum suggest that four different clusters (lobules of the posterior region, lobules of the anterior cerebellum, lobules of the vermis and lobules of left/right Crus I) may account for a significant portion of variance in the cerebellar anatomy^58^. Thus, Crus I and the vermis belong to two different clusters. During development, the territory that will generate the cerebellum is allocated during the early embryonic segmental phase of hindbrain development^59^. This suggests that development of the vermis (a medial expansion of the cerebellar hemisphere) is closely related to the expansion of the Crus I region. As ASD is considered as an early neurodevelopmental disorder, abnormal development in one cerebellar region such as Crus I may affect other regions such as the vermis. A similar pattern of a reduced Crus I but increased vermis volume has been previously find in children with ASD^2^. Since we found a correlation between cerebellar anatomy and fixation time to the eyes in patients but not among controls, one hypothesis may be that the cerebellar neurodevelopmental abnormalities are related to the abnormal development of eye contact in individuals with ASD.

There is evidence that the dorsal cerebellar vermis (lobule VI and VII) is also involved in oculomotor control. After the ablation of the dorsal vermis, Takagi *et al*.^60^ reported that monkeys presented altered saccades. In individuals with ASD, our group^61^ showed that individuals with ASD performing the Step/Overlap/Gap paradigm had, compared to controls, a reduced gain and peak velocity as well as a greater trial-to-trial variability in task performance. However, visual orienting and attentional engagement were preserved in individuals with ASD. Similarly, Schmitt *et al*.^62^ reported saccades alterations in individuals with ASD and found that visual orienting and attention system were spared. These results suggest that the subtle oculomotor abnormalities in individuals with ASD are not likely to have a major influence on the fixation time to the eyes, as investigated in our study. However, further studies are required to investigate the neuroanatomical correlates between subtle alterations in basic oculomotor tasks and cerebellar morphology in individuals with ASD.

Eye-tracking can provide stratifying biomarkers associated with cerebellar morphology and may help to better understand and investigate the heterogeneity of ASD. A limitation of neuroimaging studies in psychiatry and more particularly in the field of ASD is the heterogeneity of the patient populations, which may contribute to inconsistent findings. To help overcome this limitation, researchers may study the correlation between neuroimaging and clinical dimensions such as repetitive behaviors or the sub-scores of the ADOS scale. However, recognition is growing of the limitations of behaviorally defined categorical diagnoses for understanding the neurobiology of ASD. Moreover, we suggest that oculomotor impairments in individuals with ASD may could be used as an intermediate phenotype to better understand the pathophysiology of ASD.

We also found that the right anterior cerebellum in individuals with ASD is decreased compared with healthy controls. The anterior cerebellum is separated from the posterior lobe by the primary fissure, and it projects and receives input from the sensorimotor area. The cerebellar motor syndrome is caused by damage to the anterior lobe of the cerebellum and adjacent parts of lobule VI, where the anatomical projections to and from sensorimotor areas are located^12^. Forty-two to 88% of children with autism^63^ present a wide range of sensori-processing disorders^64^, including deficits in sensori-motor coordination, minor cerebellar motor syndrome and abnormal auditory, visual, touch and oral sensory processing^65^. Further studies are needed to investigate the relationship between the anterior cerebellar lobe in individuals with ASD and sensory-motor impairments in children including clinical scores and occulomotor tasks such as saccades and gaze fixation^62^.

In our large multicenter sample of 134 individuals with ASD and 160 controls, we did not find any difference in global cerebellar volume between adult individuals with ASD and healthy controls in the bilateral cerebellar grey or white matter. This finding is in contradiction with Yang *et al*.^66^ who found, in a meta-analysis of whole brain voxel based morphometric (VBM) studies in adults with ASD, evidence of a decreased grey matter volume in the right cerebellum. However, VBM was not originally designed for the analysis of subcortical structures and the complicated anatomy of the cerebellum may hamper the automatic processing of VBM^67^. To our knowledge, no study has specifically examined differences in cerebellar volume in adult individuals with ASD.

Several strengths support our study. First, we manually inspected each cerebellar segmentation. In the cerebellar parcellation analyses, we visually examined each slice of the cerebellum to ensure the quality of the segmentation. This quality control is key to reducing the inter-subject variability of the cerebellar shapes^68^. While this information was not available for the individuals with ASD of the ABIDE database, all individuals with ASD recruited in France had no history of alcohol abuse or dependence which is well known to affect the cerebellum^69^ and can lead to its size reduction^70,71^. Further, we only included adult individuals with ASD in our study to reduce clinical heterogeneity, which is a major limition to clinical studies and in particular neuroimaging studies^72^. Fountain *et al*.^73^ highlighted six different developmental trajectories in children with ASD. Helles *et al*.^74^ reported that 20% of adult men with a childhood diagnosis of Asperger syndrome no longer met criteria for a clinical ASD diagnosis and showed major improvements in general functioning. Clinical studies in children with ASD are likely to include heterogeneous individuals with ASD with potentially variable outcome in adult age. Studying adult individuals with ASD is important and interesting because this population is likely to have a stable diagnosis across the lifespan^75^.

Several limitations should be considered before interpreting our results. We were only able to examine the sub-volumes of the cerebellum for subjects recruited in France because of the heterogeneous quality of the MRI included from the ABIDE database and because only a subset of 59 subjects underwent an eye-tracking assessment. Thus, there is a need to replicate our findings in a larger sample, particularly the correlation between eye-tracking and cerebellar sub-volume Moreover, our finding of decreased volume in the anterior cerebellum in patients highlights the need to further investigate motor symptoms in ASD^76^, as the anterior cerebellum is connected to the sensory motor-cortex.

Our study is the first to report a link between fixation time to the eyes, which is a promising biomarker of ASD, and the morphology of the cerebellum, a brain region highly implicated in the disorder^14^.

Our results suggest that subtle changes in cerebellar sub-volumes in individuals with ASD may be related to eye avoidance or impairments in social interaction and communication.

## Electronic supplementary material

Supplementary Material
