# Supplementary material for: Cerebellar anatomical alterations and attention to eyes in autism
Source: Sci Rep. 2017 Sep 20;7:12008. doi: 10.1038/s41598-017-11883-w (PMC5607223; doi:10.1038/s41598-017-11883-w)

**Supplementary Material**

**Cerebellar anatomical alterations and attention to eyes in autism**

Charles Laidi^1,2,3,4^, Jennifer Boisgontier^1,2,3^, M. Mallar Chakravarty ^5,6^, Sevan Hotier^1,2,3,4^, Marc-Antoine d’Albis^1,2,3,4^, Jean-François Mangin^7^, Gabriel Devenyi^5^, Richard Delorme^3,8,9^, Federico Bolognani ^10^, Christian Czech^10^, Céline Bouquet^10^, Elie Toledano^10^, Manuel Bouvard^11^, Dorian Gras^12^, Julie Petit^8^, Marina Mishchenko^13^, Alexandru Gaman^2,3,4^, Isabelle Scheid^2,3,4,8^, Marion Leboyer^2,3,4,14^, Tiziana Zalla^3,13^ Josselin Houenou^1,2,3,4,5^.

1: UNIACT, Psychiatry Team, Neurospin Neuroimaging platform, CEA Saclay, (Gif-Sur-Yvette, France). 2: Institut National de la Santé et de la Recherche Médicale (INSERM), U955, Institut Mondor de Recherche Biomédicale, Psychiatrie Translationnelle (Créteil, France). 3: Fondation Fondamental (Créteil, France). 4: Pôle de Psychiatrie, Assistance Publique–Hôpitaux de Paris (AP-HP), Faculté de Médecine de Créteil, DHU PePsy, Hôpitaux Universitaires Mondor (Créteil, France). 5: Cerebral Imaging Center, Douglas Mental Health University, McGill University (Montréal, Canada). 6 : Departments of Psychiatry and Biological and Biomedical Engineering, McGill University. 7: UNATI, Neurospin neuroimaging platform, CEA Saclay, (Gif-Sur-Yvette, France). 8: Service de psychiatrie de l'enfant et de l'adolescent, Assistance Publique–Hôpitaux de Paris (AP-HP), Hôpital Robert Debré (Paris, France). 9: Institut Pasteur, Human Genetics and Cognitive Functions Unit, Paris, France. 10: Neuroscience, Ophthalmology, and Rare Diseases (NORD), Roche Pharma Research and Early Development, Roche Innovation Center Basel, F. Hoffmann-La Roche Ltd. 11: Charles Perrens Hospital, Autism Expert Center, Bordeaux, France. 12: Laboratoire de Linguistique Formelle UMR 7110, Centre National de Recherche Scientifique, Université Paris Diderot (Paris, France) 13: Institut Jean Nicod, Centre National de la Recherche Scientifique, Ecole Normale Supérieure (Paris, France). 14: Faculté de Médecine, Universite Paris Est (Créteil, France).

**Supplementary material 1**

1. Cerebellar parcellation in the ABIDE sample

We did not perform cerebellar parcellation in the ABIDE sample due to a technical problem: when using the SUIT pipeline, the cerebellum was not well isolated from the rest of the brain in the ABIDE sample. In addition, three groups of subjects (NYU, USM and CAL) yielded many parcellation errors independently from the isolation issues. Last, removing many subjects because of parcellation defects would have led to a diminution of the sample of each site, and we wanted to maintain sample size above ten in order to minimize the site effect. These segmentations issues may be partly related to the quality of the MRI scans ^42^ (https://spectrumnews.org/news/imaging-database-autism-aims-outgrow-quality-concerns/) included in the ABIDE dataset (data are shared regardless of movement artifacts) ^34^.

B. Quality assessment of cerebellar parcellation


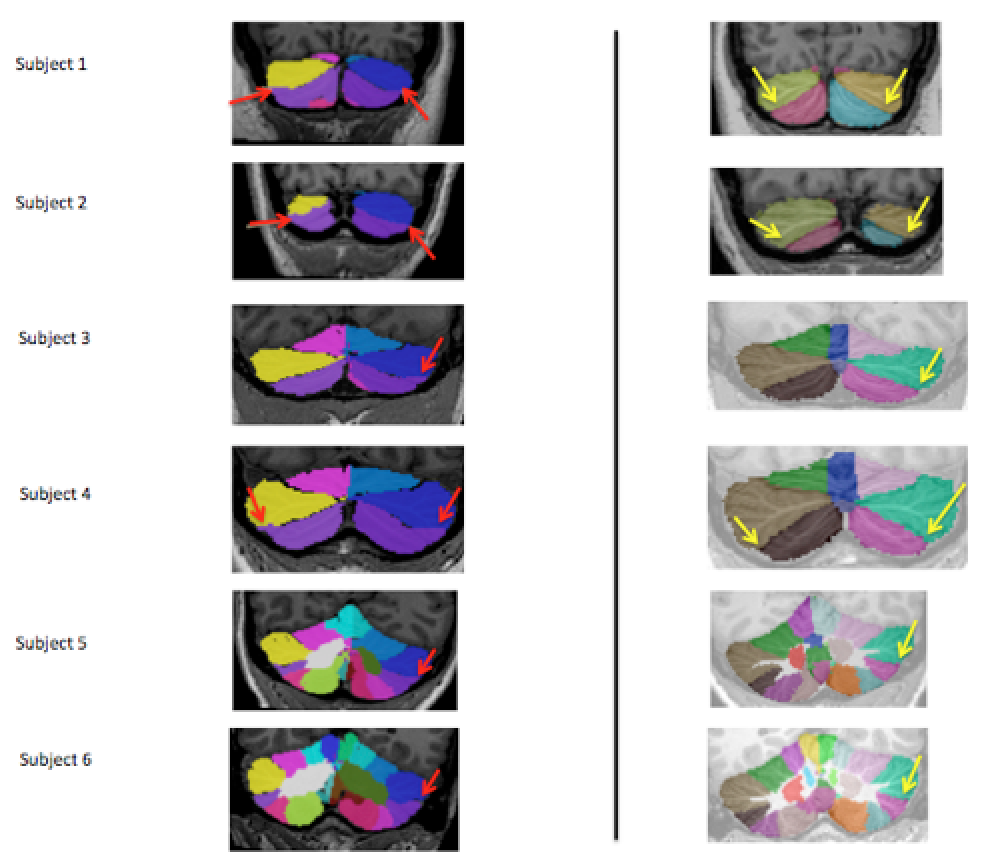


**Supplementary material 2**

Demographic and clinical characteristics of participants with eye-tracking measures

|  | ASD (n= 33) | Control (n= 26) | Chi-2 / t-test |
| --- | --- | --- | --- |
| Mean age (SD) | 30 (9.5) | 30 (8.4) | Student t-test; p = 0.8 |
| Sex: Men (%) | 25 (75) | 18 (60) | Chi-2; p = 0.39 |
| Intracranial volumes cm3 (SD) | 1.54 x 10^6^ (2.0 x 10^5^) | 1.54 x 10^6^ (2.0 x 10^5^) | Student t-test; p = 0.42 |
| Mean full scale IQ (SD) | 104 (18) | 109 (16) | Student t-test; p = 0.3 |
| Mean ADOS scores (SD) | 11 (4) | N/A | N/A |
| Fixations time to the eyes in ms (SD) | 524.93 (529) | 591.11 (416) | Student t-test; p = 0.59 |

**Supplementary material 3**

Patients and controls underwent a free-viewing eye-tracking task using a screen-based (23”, resolution set at 1920x1080 pixels) Tobii TX300 device (Tobii Technology, Stockholm, Sweden), allowing gaze acquisition at 300 Hz.

Participants were positioned 60 cm in front of a screen of 22'', with their head positioned on a chinrest in a dark room. Stimuli consisted of a set of 3 seconds lasting video of avatars (with a balanced proportion of men an women) expressing three types of facial emotions (happiness, fear, anger) with two different intensities (low or high) for a total of 18 dynamic avatar faces. An area of interest on the eyes of the actor was defined automatically. It was delimited in a rectangle encompassing both eyes. Each video showed dynamically the 3 types of facial emotions, starting with a neutral face (see Figure below).

During each trial, the sum of all the fixations on the area of interest ≥ 60 ms was calculated. The mean time of fixation on the eyes was then obtained across all trials.


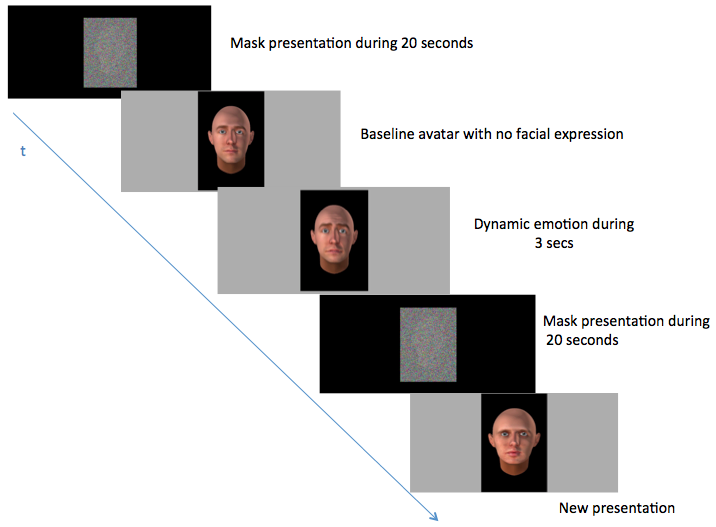


Avatar were created with FACSGen software developped by the Swiss Center for Affective Neuroscience (Modified from Roesch et al. ^79^ with copyright holder permission)

**Supplementary material 4**

Raw correlation between the fixation time to the eyes region and cerebellar sub volumes in individuals with FASD (scatter plots)

A. Vermis


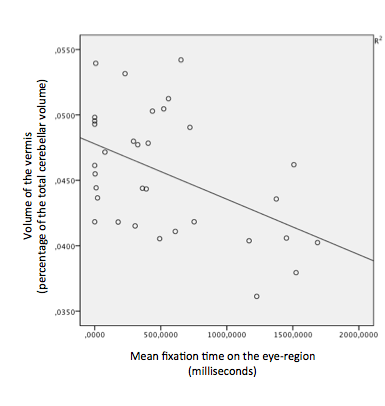


B. Left Crus I


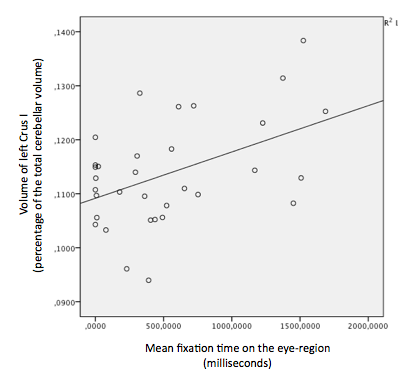


C. Right Crus I


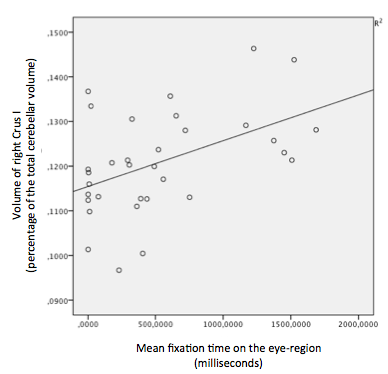


Raw correlation between the fixation time to the eyes region and cerebellar sub volumes in controls (scatter plots)

1. Vermis


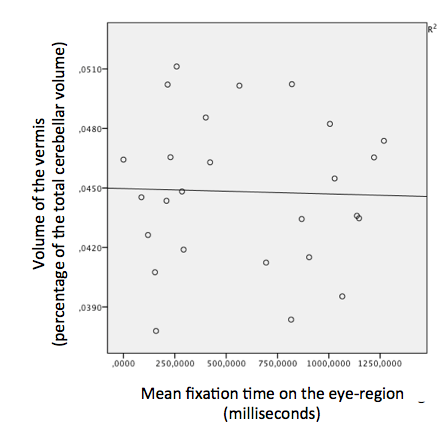


B. Left Crus I


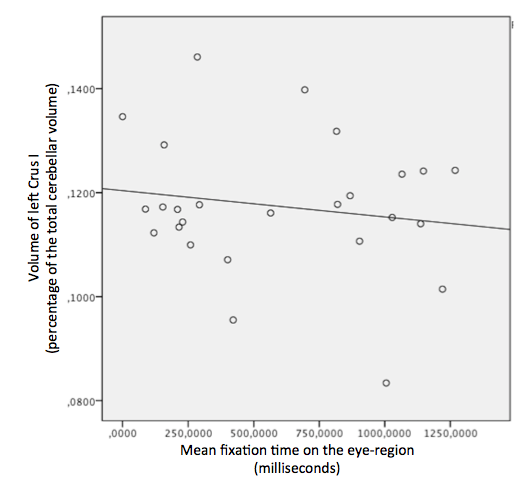


C. Right Crus I


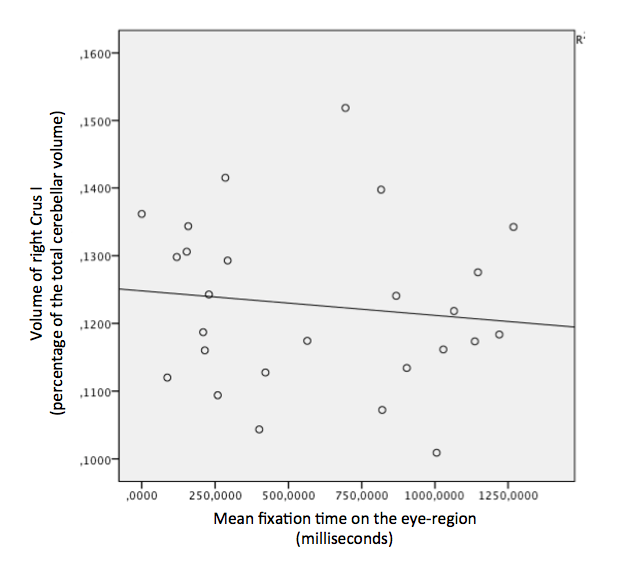

Supplement: Supplementary file 1 — Supplementary Material [file 41598_2017_11883_MOESM1_ESM.doc]
